# Magnetic resonance imaging of the breast: role in the evaluation of ductal carcinoma in situ

**DOI:** 10.1590/0100-3984.2018.0058

**Published:** 2019

**Authors:** Carla Chizuru Tajima, Luiza Lourenço Campos de Sousa, Gustavo Lagreca Venys, Camila Souza Guatelli, Almir Galvão Vieira Bitencourt, Elvira Ferreira Marques

**Affiliations:** 1 Departamento de Imagem – A.C.Camargo Cancer Center, São Paulo, SP, Brazil.

**Keywords:** Radiology, Carcinoma, intraductal, noninfiltrating, Magnetic resonance imaging, Breast neoplasms, Radiologia, Carcinoma intraductal não infiltrante, Ressonância magnética, Neoplasias da mama

## Abstract

Ductal carcinoma in situ (DCIS) is a precursor mammary lesion whose malignant cells do not extend beyond the basement membrane and presents a risk of progression to malignant disease. Its early detection increased with screening mammography. The objective of this study was to review the literature on the main presentations of DCIS on magnetic resonance imaging (MRI), through searches of the Medline/PubMed, Latin-American and Caribbean Center on Health Sciences Information (Lilacs), and Scientific Electronic Library Online (SciELO) databases. DCIS can occur in its pure form or in conjunction with invasive disease, in the same lesion, in different foci, or in the contralateral breast. MRI has a high sensitivity for the detection of pure DCIS, being able to identify the non-calcified component, and its accuracy increases with the nuclear grade of the lesion. The most common pattern of presentation is non-nodular enhancement; heterogeneous internal structures; a kinetic curve showing washout or plateau enhancement; segmental distribution; and restricted diffusion. MRI plays an important role in the detection of DCIS, especially in the evaluation of its extent, contributing to more reliable surgical excision and reducing local recurrence.

## Abstract (pt)

O carcinoma ductal *in situ* (CDIS) é uma lesão mamária precursora cujas células malignas não ultrapassam a membrana basal e possui risco de evolução para doença maligna. Sua detecção precoce aumentou com a mamografia de rastreamento. O objetivo deste estudo foi realizar uma revisão da literatura sobre as principais apresentações do CDIS na ressonância magnética (RM), utilizando mecanismos de busca na base de dados Medline/PubMed, Centro Latino-Americano e do Caribe de Informação em Ciências da Saúde (Lilacs) e Scientific Electronic Library Online (SciELO). O CDIS pode ocorrer de forma pura ou associado a doença invasiva, na mesma lesão, em focos diferentes e na mama contralateral. A RM possui alta sensibilidade para a detecção do CDIS puro, sendo capaz de identificar o componente não calcificado da doença e sua precisão aumenta com o seu grau nuclear. O padrão de apresentação mais comum é o realce não nodular com padrão interno heterogêneo, curva cinética de lavagem rápida (*washout*) ou platô, de distribuição segmentar e com restrição na difusão. A RM tem importante papel na detecção do CDIS e, principalmente, na avaliação da sua extensão, contribuindo para uma exérese cirúrgica mais confiável e reduzindo as recidivas locais.

## INTRODUCTION

Breast cancer is the most common malignant neoplasm among women and the second leading type of cancer worldwide. According to the Brazilian National Institute of Cancer, breast cancer accounts for approximately 25% of all cases of cancer in Brazil^(^1^-^3^)^.

Ductal carcinoma *in situ* (DCIS) is a heterogeneous disease consisting of malignant epithelial cells that originate in the terminal ductal lobular and do not cross the basement membrane. It is considered a precursor lesion and presents a risk of developing into invasive mammary neoplasia^(^4^-^6^)^. Early detection of DCIS has increased significantly with the use of screening mammography in women over 40 years of age, a practice that was put in place in the mid-1980s and has a reported sensitivity of 60-90%^(^7^,^^8^^)^.

Magnetic resonance imaging (MRI) of the breasts, which has been widely used since the 1990s, allows us to distinguish normal tissue from cancerous tissue through identification of the increased vascularity and capillary permeability of malignant lesions and can be considered complementary to mammography, especially in the evaluation of disease with no calcifications and of the extent of a tumor^(^2^,^^6^^)^. MRI has high sensitivity for the diagnosis of DCIS, especially for high-grade tumors^(^5^)^.

The objective of this study was to discuss and illustrate the ways in which DCIS can present on an MRI scan. We also review the effectiveness of MRI in the early detection and evaluation of the extent of the disease.

## MATERIALS AND METHODS

We selected English-language articles presenting imaging examinations in the evaluation of DCIS. In our initial searches-conducted in the databases operated by Medline/PubMed, Latin-American and Caribbean Center on Health Sciences Information (LILACS), and Scientific Electronic Library Online (SciELO)-we used the following search terms: DCIS; image; and breast MRI. We limited our searches to articles published between 2000 and 2018. We thus identified 28 articles that presented descriptive information related to the radiological aspects of DCIS, including those seen on MRI. In addition, we evaluated the imaging findings for patients diagnosed with DCIS and undergoing MRI at a referral center for cancer.

## DISCUSSION

### Clinical and pathological aspects

DCIS is a heterogeneous lesion that is considered a pre-invasive form of breast cancer; it is the most common noninvasive type. It has a high potential for progression to invasive disease and does so in approximately 30% to 50% of cases^(^6^)^, especially those of one of the subtypes with a high nuclear grade, which can progress to invasive disease and require surgical treatment. Clinically, DCIS is asymptomatic in most patients, being an incidental finding in routine imaging examinations, mainly being identified by microcalcifications in the mammography. With the advent of mammographic screening programs and improvements in the quality of imaging examinations, the rate of early detection of DCIS has increased by approximately 20%^(^5^)^.

The morphological classification of DCIS by nuclear grade divides tumors into the following groups: low-grade, intermediate-grade, and high-grade. Low-grade tumors are well differentiated and show no necrosis; intermediate-grade tumors are well or moderately differentiated and have some areas of necrosis; and high-grade tumors are poorly differentiated, present areas of necrosis, and have a high level of cellular proliferation. The architectural subtypes include the pattern types cribriform, micropapillary, solid, mixed, and comedo. Comedo-DCIS has the worst prognosis, with a large number of atypical cells and an extensive area with necrotic debris, surrounded by a layer of cells also with atypia, often with microcalcifications and numerous mitoses^(^9^-^11^)^. A high nuclear grade of DCIS is associated with a worse prognosis and, when accompanied by comedonecrosis, can correlate with a higher risk of local recurrence after surgical excision.

### Presentation of DCIS in conventional methods (mammography and ultrasound)

**Figure 1:**
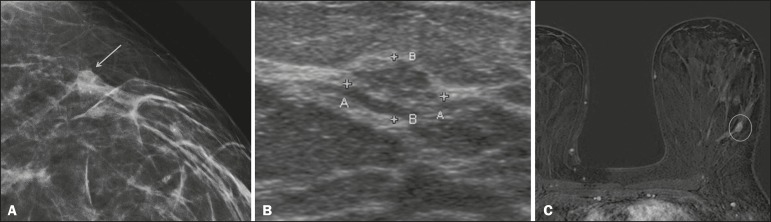
A: Mammography, with focal craniocaudal compression, showing an ovoid nodular image with irregular margins, containing amorphous calcifications, in the posterior third of the upper outer quadrant of the left breast (arrow). B: Ultrasound of the left breast showing an ovoid nodular image, with indistinct margins, containing echogenic areas suggestive of microcalcifications, located between the two o’clock and three o’clock positions on the left breast, corresponding to the nodular image in the upper outer quadrant described in the mammographic examination. C: Axial MRI sequence of the breasts, with digital subtraction, showing an irregular nodule in the upper outer quadrant of the left breast. The pathology study revealed DCIS, solid and cribriform types, nuclear grades 2 and 3, with foci of apocrine differentiation.

In most mammography images, DCIS presents as microcalcifications of varying morphologies, such as amorphous, coarse, heterogeneous, or fine pleomorphic. The fine pleomorphic morphology creates the highest suspicion for high-grade lesions. The distribution of microcalcifications in the breast varies among the grouped, linear, and segmental forms (Figure 1). A smaller proportion appear as masses or areas of architectural distortion^(^11^,^^12^^)^.

An ultrasound examination can be useful in the detection of DCIS, especially in the evaluation of masses with calcifications observed in the mammogram, increasing the specificity of this method (Figure 1B). The noncalcified area may represent the invasive component of the lesion^(^9^)^.

### Presentation of the DCIS on the MRI

**Figure 2:**
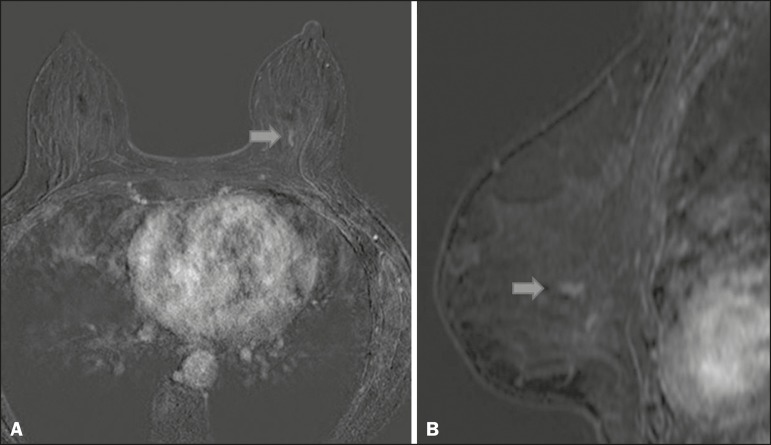
Contrast-enhanced, high-resolution MRI. Axial sequence, with digital subtraction (A) and sagittal MRI sequence (B), showing a linear area of enhancement (arrows) in the posterior third of the central region/junction of the medial quadrants of the left breast. The pathology study of the surgical specimen revealed DCIS, nuclear grade 2.

In recent years, MRI of the breasts has often been used as a complement to mammography and ultrasound. MRI shows a high sensitivity for the detection of pure DCIS or DCIS associated with invasive carcinoma (Figure 2), which is quite helpful in the assessment of the noncalcified component of the disease^(^11^,^^13^^)^, as well as in the evaluation of tumor extent and of residual disease; in the identification of an occult primary tumor; in the detection of multifocal, multicentric, and contralateral tumors; in the evaluation of the response to neoadjuvant chemotherapy; in preoperative staging; and in the evaluation of any inconclusive findings of mammographic examinations.

MRI is useful in the detection of DCIS, especially high-grade DCIS, even in cases in which the mammogram is normal. The sensitivity of MRI for the detection of DCIS varies widely, from 60% to 100%, especially when high-resolution sequences are acquired, and may be useful for calcified or noncalcified carcinomas^(^10^,^^14^^-^17^)^. Pure DCIS lesions show non-nodular enhancement in 59% of cases, whereas 14% enhance as a nodule, 14% do not enhance, and 12% present as a focus^(^18^)^. In contrast, 76% of the lesions associated with an invasive carcinoma and DCIS enhance as a nodule^(^14^)^.

In another study of DCIS, Kuhl et al.^(^5^)^ demonstrated that the sensitivity of MRI was far superior to that of mammography for the detection of DCIS (92% vs. 56%). Those authors also found that most (87%) of the lesions not detected in MRI were low-grade tumors. Improvements in the detection of DCIS reported in recent studies are probably due to improvements in the spatial and temporal resolution used in high-resolution MRI sequences^(^5^)^. Another recent study showed that the sensitivity of MRI is superior to that of conventional methods for the diagnosis of low-grade DCIS (74.0% vs. 40.7%), intermediate-grade DCIS (84.1% vs. 34.9%), and high-grade DCIS (91.8% vs. 36.7%), the difference being greatest in the last group^(^19^)^.

**Figure 3:**
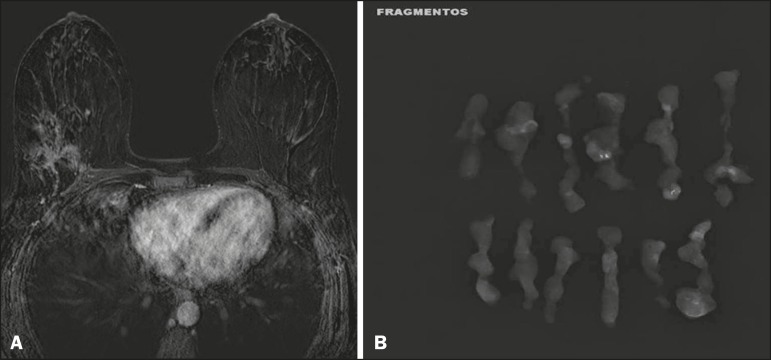
High-resolution MRI of the breasts. A: Axial sequence, with digital subtraction, showing an area of non-nodular enhancement with segmental distribution, with a heterogeneous pattern within the area, which was located in the upper outer quadrant/junction of the lateral quadrants of the right breast, corresponding to the area of fine pleomorphic microcalcifications of segmental distribution described in the mammographic examination. B: The image shows fragments from the vacuum-assisted, stereotactically-guided percutaneous biopsy, with microcalcifications. The pathology study revealed DCIS of the cribriform, comedo, and micropapillary types, nuclear grade 3, extending to the lobules. Note the comedonecrosis.

**Figure 4:**
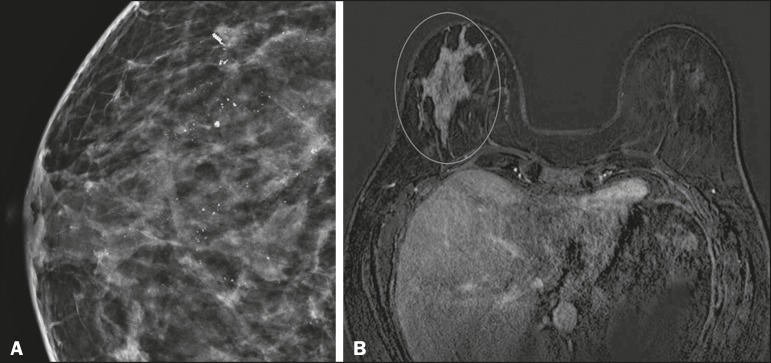
A: Magnified craniocaudal mammography showing extensive grouping of grossly heterogeneous microcalcifications with segmental distribution in the right breast, together with a metallic clip (tissue marker) placed during the biopsy. B: Axial MRI sequence of the breasts, with digital subtraction, showing an area of heterogeneous non-nodular enhancement with segmental distribution and minimal post-contrast enhancement, occupying the lower outer quadrant of the right breast, corresponding to the findings described in the mammographic examination. The patient underwent mastectomy, and the pathology study revealed DCIS, nuclear grade 3, invading the lobules.

Although the most common pattern of presentation of DCIS on MRI is non-nodular enhancement, it can also present as a mass or focus. The types of distribution vary among segmental, linear/ductal, focal, diffuse, and regional. High-grade DCIS typically manifests as areas of non-nodular enhancement (in 60-81% of cases) with a heterogeneous internal pattern and segmental distribution^(^20^)^, as depicted in Figures 3 and 4.

The kinetic curves can be classified as follows^(^6^)^: persistent (type I), with a signal that increases over time; plateau (type II), in which the intensity of the signal does not change; or wash-out (type III), with a drop in signal over time. The types II and III are the most common curves in DCIS.

Pure (noncalcified) DCIS can be symptomatic, especially in patients with dense breasts (in whom the mammographic assessment is more difficult), presenting as palpable masses or as a complaint of papillary discharge. On a mammogram, it can present as focal asymmetries or architectural distortion, whereas, on ultrasound, it can present as hypoechoic masses with indistinct, angular, or spiculated margins. On MRI, its presentation varies, although it typically presents as a nodule and non-nodular enhancement, type II and III curves prevailing. Although there is no consensus regarding the nuclear grade of noncalcified DCIS, it has been suggested that it is most often of a high nuclear grade with comedonecrosis^(^21^,^^22^^)^.

**Figure 5:**
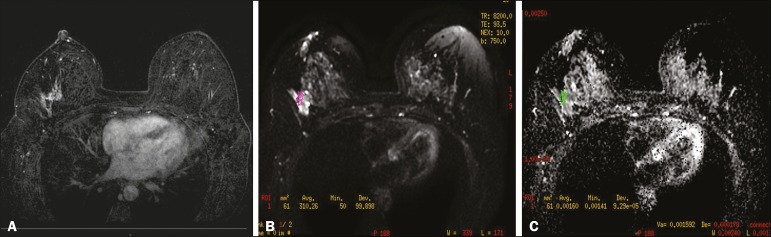
Axial MRI sequence of the breasts, with digital subtraction (A), showing an area of heterogeneous non-nodular enhancement with segmental distribution, located in the posterior third of the upper outer quadrant of the right breast. The image in B shows restricted diffusion in the ADC map (C), the ADC value being 1.6 × 10^−3^ mm^2^/s. The patient was submitted to quadrantectomy, and the pathology study revealed DCIS, nuclear grade 1, with atypical ductal hyperplasia.

The diffusion-weighted imaging sequence, widely used in the evaluation of intracranial diseases since the 1990s, can also be used for the evaluation of extracranial changes. On the basis of the random motion of water molecules in biological tissues, the apparent diffusion coefficient (ADC) can be calculated^(^23^)^. In breast tumors, cell proliferation restricts the movement of water molecules, reducing the ADC values^(^24^,^^25^^)^. Studies have shown that DCIS has ADC values below those of normal fibroglandular tissue and significantly above those of invasive ductal carcinoma^(^26^)^. However, DCIS is a heterogeneous lesion, with high- and low-grade components coexisting within a single lesion, the classification of which is usually determined by the component of the highest grade (Figure 5).

Gauging the extent of the lesion is fundamental to treatment planning and to reducing the risk of disease recurrence, and studies have shown that MRI is more reliable than is mammography in evaluating the size of DCIS, contributing to a better surgical result with the removal of the affected area without compromised margins and, consequently, to a lower chance of local recurrence^(^13^,^^21^^-^26^)^. MRI can also show the noninvasive component in invasive carcinomas diagnosed by other methods, contributing to a better definition of the true extent of the disease^(^20^)^. The presence of an extensive intraductal component in invasive carcinoma is associated with a worse prognosis and greater overestimation of the size of the tumor on MRI, when compared with the pathological evaluation, which includes only the invasive component^(^26^-^28^)^.

## CONCLUSION

The presentation of a DCIS in imaging examinations can vary greatly, creating a dilemma for the radiologist. Therefore, MRI plays an important role in the detection of DCIS, especially in evaluating its extent, thus making surgical excision more reliable and reducing the rate of local recurrence. Therefore, it is essential that radiologists recognize its main presentations and use complementary MRI resources, such as the analysis of kinetic curves and diffusion-weighted sequences. Thus, radiologists will be able to make the correlation with other imaging methods, such as mammography and ultrasound, in order to detect the disease earlier and initiate the appropriate treatment in a timely manner.
